# Genomic imbalance of *HMMR/RHAMM* regulates the sensitivity and response of malignant peripheral nerve sheath tumour cells to aurora kinase inhibition

**DOI:** 10.18632/oncotarget.793

**Published:** 2013-01-09

**Authors:** Pooja Mohan, Joan Castellsague, Jihong Jiang, Kristi Allen, Helen Chen, Oksana Nemirovsky, Melanie Spyra, Kaiji Hu, Lan Kluwe, Miguel Angel Pujana, Alberto Villanueva, Victor F. Mautner, Jonathan J. Keats, Sandra E. Dunn, Conxi Lazaro, Christopher A. Maxwell

**Affiliations:** ^1^ Department of Pediatrics, Child & Family Research Institute, University of British Columbia, Vancouver, British Columbia, Canada; ^2^ Hereditary Cancer Program, Catalan Institute of Oncology, Bellvitge Institute for Biomedical Research, L'Hospitalet de Llobregat, Barcelona, Spain; ^3^ Integrated Cancer Genomics Division, The Translational Genomics Research Institute, Phoenix, AZ, USA; ^4^ Department of Neurology, University Hospital Eppendorf, Hamburg, Germany; ^5^ Translational Research Laboratory, Catalan Institute of Oncology, Bellvitge Institute for Biomedical Research, L'Hospitalet de Llobregat, Barcelona, Spain

**Keywords:** MPNST, AURKA, RHAMM, TPX2, cancer stem cell

## Abstract

Malignant peripheral nerve sheath tumours (MPNST) are rare, hereditary cancers associated with neurofibromatosis type I. MPNSTs lack effective treatment options as they often resist chemotherapies and have high rates of disease recurrence. Aurora kinase A (AURKA) is an emerging target in cancer and an aurora kinase inhibitor (AKI), termed MLN8237, shows promise against MPNST cell lines *in vitro* and *in vivo*. Here, we test MLN8237 against two primary human MPNST grown *in vivo* as xenotransplants and find that treatment results in tumour cells exiting the cell cycle and undergoing endoreduplication, which cumulates in stabilized disease. Targeted therapies can often fail in the clinic due to insufficient knowledge about factors that determine tumour susceptibilities, so we turned to three MPNST cell-lines to further study and modulate the cellular responses to AKI. We find that the sensitivity of cell-lines with amplification of *AURKA* depends upon the activity of the kinase, which correlates with the expression of the regulatory gene products TPX2 and *HMMR*/RHAMM. Silencing of *HMMR*/RHAMM, but not TPX2, augments AURKA activity and sensitizes MPNST cells to AKI. Furthermore, we find that AURKA activity is critical to the propagation and self-renewal of sphere-enriched MPNST cancer stem-like cells. AKI treatment significantly reduces the formation of spheroids, attenuates the self-renewal of spheroid forming cells, and promotes their differentiation. Moreover, silencing of *HMMR*/RHAMM is sufficient to endow MPNST cells with an ability to form and maintain sphere culture. Collectively, our data indicate that AURKA is a rationale therapeutic target for MPNST and tumour cell responses to AKI, which include differentiation, are modulated by the abundance of *HMMR*/RHAMM.

## INTRODUCTION

Neurofibromatosis type I is an autosomal dominant, tumour predisposition syndrome [1]. The disease affects about 1 in 3000 young adults and causes the occurrence of benign dermal and plexiform tumours. Approximately 8-13% of affected individuals will develop malignant peripheral nerve sheath tumours (MPNSTs) in their lifetime [2]. Sporadic MPNST are rare in the general population but account for approximately half of diagnosed MPNST cases [3]. Both sporadic and NF1-associated MPNST are difficult to treat owing to their resistance to chemotherapy and frequent deep seated location in the body, which results in 5-year survival rates in the range of 20-50% [2-4].

The molecular mechanisms underlying the progression of MPNSTs remain unclear, which limits the discovery and application of new targeted therapies. Patel and colleagues identified Aurora kinase A (AURKA) as a candidate target for MPNST therapy by first modeling the disease in an animal model, identifying frequent amplification of *AURKA* in human tumours, and then treating MPNST cell-lines grown *in vitro* and as xenotransplants *in vivo* with an AURKA specific inhibitor, termed MLN8237 [5]. AURKA is an oncogenic kinase that enables mitotic spindle assembly [6], and aurora kinase A inhibitors (AKI) often induce a G2/M cell cycle arrest followed by apoptosis in cancer cell lines grown *in vitro* and *in vivo* [7, 8]. Inhibitors of Aurora kinase B (AURKB) are also effective anti-mitotic drugs, which often induce mitotic failure and endoreduplication [8]. Treatment of an MPNST cell-line with MLN8237 stabilized the cell-line's growth *in vivo* and induced endoreduplication and senescence *in vitro* [5]. Optimal application of these emerging therapies will require a better understanding and prediction of MPNST susceptibilities and tumour cell responses.

The examination of copy number variants in human, primary dermal or plexiform neurofibromas and MPNSTs of differing grades provides an additional link between disease progression and the AURKA signalling pathway. In approximately half of the high-grade tumours and not in low-grade MPNSTs or neurofibromas, hemizygous deletion of the *hyaluronan mediated motility receptor (HMMR)* gene was reported [9]. The *HMMR* gene encodes a multifunctional protein (RHAMM) that enables mitotic spindle assembly [10] and may attenuate the activation of AURKA [11], which relies upon a heterodimeric complex with targeting protein for XKLP2 (TPX2) [12]. As RHAMM binds TPX2 [13, 14], the hemizygous loss of *HMMR*/RHAMM that is often associated with the progression of MPNSTs may release TPX2, activate AURKA, and provide a growth advantage. Indeed, both *AURKA* (20q13.2) and *TPX2* (20q11.2) lie within chromosomal regions that are frequently amplified in sporadic MPNSTs [15, 16]. Taken together, these genomic imbalances may oncogene-addict high-grade MPNSTs to AURKA activity and sensitize them to the treatment with AKIs.

AURKA also regulates non-mitotic events, such as apicobasal polarization of epithelia [11], cilia resorption [17], and embryonic stem cell fate [18, 19]. In mouse embryonic stem cells (mESCs), AURKA is essential for maintenance of stem cell populations and silencing *AURKA* or treatment with AKI was sufficient to drive their differentiation through mesoderm and ectoderm lineages [18]. Thus, AKI may have effects on non-mitotic processes in tumour cells, such as the determination of cell fate and differentiation.

Here, we propose that AURKA represents a rational therapeutic target for MPNSTs and that the sensitivity of these tumours to AKI may be regulated by gene dosage and expression of *HMMR*/RHAMM. We find that treatment with an AKI efficiently stabilizes the growth of two primary human MPNSTs grown as xenografts *in vivo*. The sensitivity of immortalized MPNST cell-lines to this class of drugs relies in part on the activity of AURKA, which can be modified by targeted silencing of key kinase regulators, such as *TPX2* and *HMMR*/ RHAMM. Treatment with MLN8237 also engages a differentiation program in sphere-enriched MPNST cells that impairs their self-renewal and favours differentiation with expression of neuron-specific microtubule architecture.

## RESULTS

### Aurora kinase inhibition is an encouraging preclinical treatment for human MPNST

**Figure 1:**
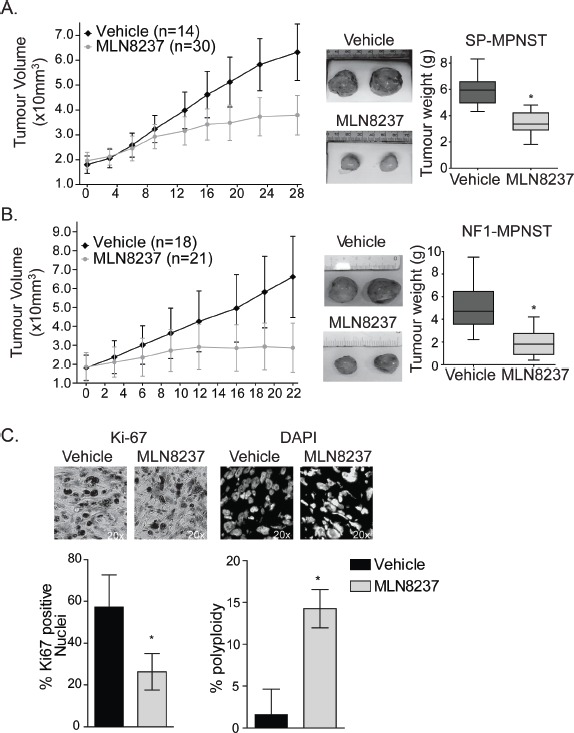
MLN8237 is effective against primary MPNSTs grown as xenotransplants in vivo A. Sporadic MPNST tumours were orthotopically transplanted into *NOD/SCID* mice and allowed to grow to 2000 mm^3^ before treatment of 30mg/kg MLN8237 or vehicle was delivered daily. Treatment with MLN8237 resulted in stable disease after 2 weeks treatment, as determined by calliper measurements of tumour volumes, n=30 MLN8237 treated and n=14 for vehicle treated, *p<0.01, error bars = SD. Representative images of SP-MPNST tumours after 28 days of MLN8237 treatment or vehicle control are shown and treatment with the AKI resulted in significant reduction in tumour weight (quantitation on the right) (*p<0.01, Error bars = SD).*B.* NF1-MPNST tumours also show significantly stabilized tumour volumes and reduced tumour weight in the MLN8237 treated tumours compared to the vehicle controls. Vehicle treated mice, n=18, MLN8237 treated mice, n=21, error bars = SD. *C.* After 28 days of treatment, SP-MPNST tumour sections were stained for Ki-67 and DAPI. Ki-67 staining was significantly decreased in treated tumours suggesting that these cells have exited the cell cycle. Furthermore, there was a significant increase in multi-nucleate cells in treated tumours suggesting that cells are undergoing G2/M arrests and endoreduplication post treatment. *p<0.01, error bars = SD. Images are taken at 20x magnification.

A preclinical study of MLN8237 has shown efficacy against an immortalized MPNST cell-line that was grown *in vitro* and as a xenotransplant *in vivo* [5]. To build on these findings and test the efficacy of this new putative therapy against primary human MPNSTs, we treated two human tumours grown as explants *in vivo* (Castellsagué et al., manuscript under preparation). One sporadic (SP-MPNST) and one hereditary (NF1-MPNST) primary, human MPNST were separately transplanted orthotopically and expanded in the flank of NOD-SCID mice (n= 44 and 39, respectively) to a size of 2000 mm^3^, randomized, and treated by oral gavage with vehicle control or 30 mg/kg/day MLN8237 for a period of four weeks, which is a dosing regimen based upon the published *in vivo* pharmacodynamics for the drug [7]. Treatment of animals with MLN8237 resulted in stabilized disease for tumour explants from both patients, as opposed to the linear expansion of tumour volumes in the vehicle treated cohorts (SP-MPNST, p <0.0001; NF1-MPNST, p= 0.0011) (Fig 1A,B). Once the dosing schedule was completed, tumours were excised and weighed. Consistent with the caliper measurements, treatment with MLN8237 resulted in tumour masses that were significantly lower in both the NF1-MPNST and SP-MPNST explants (p<0.01) compared to those of vehicle treated tumours (Fig 1A,B).

For insight into the mechanisms through which MLN8237 stabilized tumour growth, we examined the excised SP-MPNSTs by immunohistochemistry and found evidence that MLN8237 causes tumour cells to exit the cell cycle and/or undergo endoreduplication. MLN8237-treated tumours contained significantly fewer Ki67positive cells than the vehicle treated controls (Fig 1C) while the fraction of polyploid cells, as measured through the DAPI counterstain, was increased within the treated tumour cells (Fig 1C).

### Immortalized MPNST cell-lines differ in their sensitivity to aurora kinase inhibitors (AKI)

**Figure 2:**
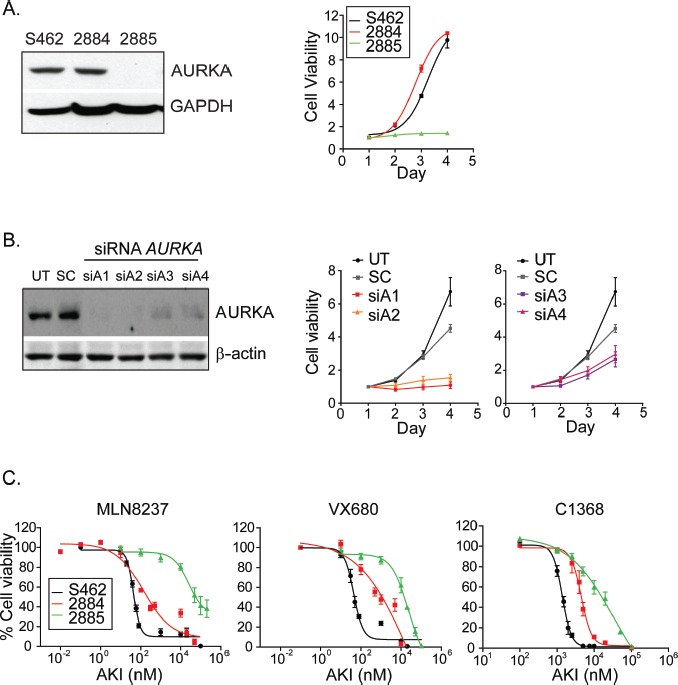
Inhibition of AURKA attenuates the growth of MPNST cell-lines in vitro *A.* Cell lysates collected from S462, 2884 and 2885 cell lines show equivalent AURKA expression in S462 and 2884 cells lines. Furthermore, these cells lines undergo equivalent growth while 2885 cells experience slow proliferation. Seeding densities for S462 and 2884 were modified to enable equivalent proliferation. Cell viability was measured by MTT assays over a 4 day period. *B.* Immunoblot analysis of AURKA expression in lysates from untreated S462 cells and those treated with scrambled or AURKA targeted siRNA at 48 hours post transfection reveal specific reduction of AURKA. β-actin serves as a loading control. Cell viability is decreased in a dose dependent manner in cells treated with siRNA targeting AURKA relative to untreated cells and those cells treated with scrambled siRNA (right). Plotted siAurora values are obtained from four redundant siRNA targeting AURKA. Error bars = SEM, n=5 replicate experiments. *C.* Treatment of MPNST cells with three inhibitors to Aurora kinases, MLN8237, VX680 and C1368, reveals marked and dose-dependent reduction in cell viability as measured by MTT after 72 hours of treatment. Error bars = SEM, n=3 replicate experiments.

To examine MPNST cellular responses to MLN8237 in greater detail, we studied three established and immortalized MPNST cell-lines. In two of the cell-lines (S462 and 2884), the expression of AURKA and the kinetics of growth *in vitro* were equivalent (Fig. 2A). The 2885 cell-line, however, was poorly proliferative and lysates from these cells contained negligible levels of AURKA (Fig. 2A). When the expression of *AURKA* was silenced in S462 cells through transfection of four distinct siRNA constructs, cell survival was significantly impacted and proportional to the degree of *AURKA* silencing achieved by the various siRNA constructs, which implied a dose-response between AURKA abundance and cell growth rate or viability (Fig. 2B). We obtained three AKIs from commercial sources and tested the ability of each AKI to reduce kinase activity. Lysates from S462 cells treated with varying concentrations of AKI showed dose dependent reduction of pRHAMM (Fig S1A). This was confirmed with immunofluorescence analysis of the auto-activated kinase (p-AURKA) [12] and two downstream substrates (pRHAMM and p-histone H3) [11, 20] (Fig S1B). Each of the MPNST cell-lines was then separately treated with each AKI. Following 72 hours of treatment with MLN8237, the survival of each MPNST cell-line *in vitro* was reduced significantly (Fig. 2C). However, the sensitivity of the cell-lines did not completely align with the levels of AURKA expression, as would be predicted from the results of the siRNA experiments.

The 2885 cell-line resisted MLN8237 treatment and required 400-725 fold higher concentrations to inhibit 50% of cellular growth (IC-50) (Fig. 2C), which is consistent with the negligible expression of AURKA in this cell-line. Despite equivalent growth rates and AURKA abundance in 2884 and S462 cells, however, the cellular responses to MLN8237 were significantly different (p=0.02) with S462 being the more sensitive (Fig. 2C). We found that S462 cells were also 2.2 – 9.2 fold more sensitive to the pan-Aurora inhibitors VX680 (IC-50- 2884, 387 ±47 nM; S462, 42 ±10 nM) and C1368 (IC-50- 2884, 2925 ±135 nM; S462, 1302 ±264 nM) (Fig. 2C), which implied that factors intrinsic to these cell-lines may influence their responses to AKI.

### Genomic imbalances in *HMMR*/RHAMM and TPX2 regulate AURKA activity and the sensitivity of MPNST cells to AKIs

**Figure 3:**
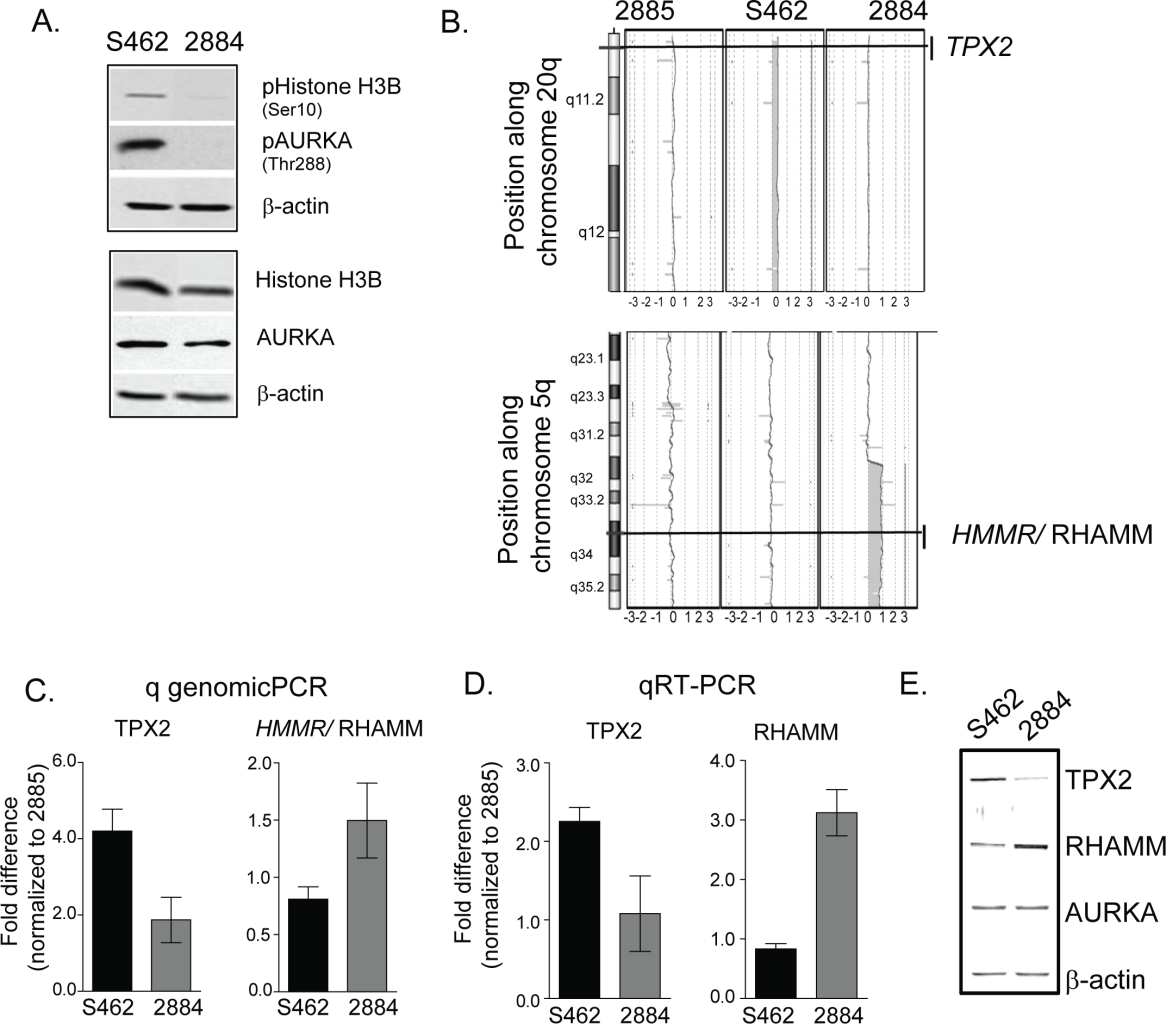
Gene dose alterations in TPX2 and RHAMM account for the differential AURKA activity in MPNST cell-lines *A.* Augmented AURKA activity is detected in S462 cell lysates as measured by immunoblot detection of pS10-Histone H3B and pT288-AURKA. Apart from an increased level of RHAMM expression in 2884, levels of the unphosphorylated proteins are relatively constant. β-actin serves as a loading control. *B.* Comparative genomic hybridization of genomic DNA from S462, 2884 and 2885 MPNST cell lines identify copy number gains of 20q in S462 and 2884 cells, and a gain of 5q33.2-qter in 2884 cells. *C.* Quantitative PCR confirms increased gene dosage of *TPX2*, but decreased *HMMR*, in S462 relative to 2884 cells. Error bars =SD, n=3. *D.* Gene dosage translates to different levels of mRNA for TPX2 and RHAMM as detected by qRT-PCR in S462 relative to 2884 cells. Error bars=SD, n=3. *E.* Representative blots for protein expression of AURKA, TPX2 and RHAMM as detected in cell lysates from S462 and 2884 cell-lines by IR labelled antibodies. Consistent with the genomic and message levels, S462 cells contain a marked increase in TPX2 expression and a reduced expression of RHAMM relative to 2884 cells.

We postulated that the sensitivity of the S462 and 2884 cell-lines to MLN8237 may reflect different levels of kinase activity, rather than AURKA abundance. Consistently, AURKA-mediated phosphorylation events were augmented in S462 cells, while the absolute levels of the substrate proteins remained relatively constant (Fig 3A). So, we screened genomic DNA from each of the MPNST cell-lines on a high density oligonucleotide a-CGH platform for insight into a putative mechanism that accounts for the elevated kinase activity in S462 cells.

The 2885 cell-line displayed a relatively diploid genome, however, we identified copy number variation in the region surrounding *AURKA* in 2884 and S462 cells (Fig. S2A, B). In S462 cells, the region surrounding *TPX2* (20q11.2) was also amplified (Fig 3B). While in 2884 cells, the region surrounding *HMMR/*RHAMM (5q33.2-qter) was amplified (Fig 3B). These genomic imbalances implied that the S462 cell-line may express more TPX2, a protein that activates AURKA [12], and less RHAMM, a protein that sequesters TPX2 [11], and these imbalances may enhance AURKA activity. With quantitative genomic PCR, we confirmed the amplification of *TPX2* and *HMMR*/RHAMM in 2884 and S462 cells, respectively (Fig. 3C). Moreover, quantitative RT-PCR (Fig 3D) and quantitative immunoblot analysis (Fig. 3E) showed that the respective gene products were expressed in these cell-lines proportionate to the underlying genomic amplifications.

**Figure 4:**
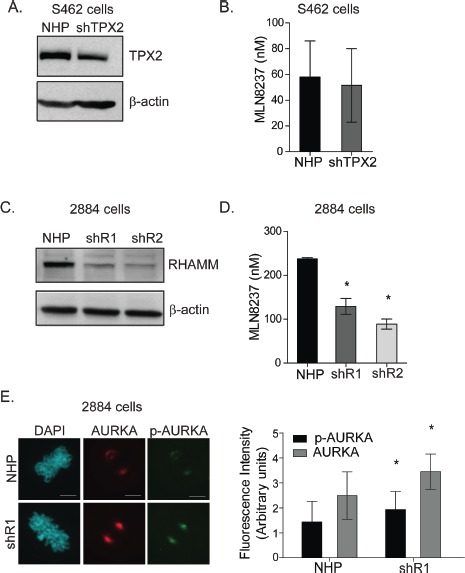
RHAMM depletion increases AURKA activity and sensitivity to AKI in 2884 cells *A*. TPX2 was stably knocked down in S462 cells using shRNA mediated silencing of either TPX2 or a control non-hairpin (NHP) construct. β-actin served as a loading control. *B.* shTPX2 S462 cells experienced no significant differences in IC-50 to MLN8237 compared to NHP cells, error bars = SD, n=3. *C.* RHAMM was knocked down in 2884 cells with a similar shRNA expression system. Two redundant shRNAs were used, shR1 and shR2. β-actin served as a loading control. *D.* shR1 and shR2 cells experienced a 2 fold decrease in IC-50 in response to MLN8237 treatment compared to NHP cells, *p value<0.05, error bars = SD, n=3. *E.* Representative images of NHP and shR1 cells at metaphase stained for AURKA and pAURKA-T288. Scale bars = 5μm. Quantitation of fluorescent intensity of spindle poles was done using FV10-ASW software, *p value <0.05, error bars = SD.

To investigate whether altering the expression of *TPX2* or *HMMR*/RHAMM is sufficient to modify MPNST cellular responses to AKI, we established stable S462 and 2884 sub-lines that express shRNA targeting *TPX2* or *HMMR*/RHAMM, respectively, as well as a non-hairpin (NHP) shRNA control. The growth kinetics for sub-lines did not differ significantly to NHP controls (Fig S3A, C). We silenced *TPX2* within S462 cells and, after drug selection, reduced TPX2 protein abundance by approximately 40% (Fig. 4A). However, the reduced expression of TPX2 did not alter the IC-50 for MLN8237 (Fig 4B) or VX680 (Fig S3B). Next, we silenced *HMMR/* RHAMM within 2884 cells with redundant constructs (shR1 and shR2) and achieved stable reduction of approximately 65% of RHAMM protein after drug selection (Fig. 4C). The IC-50 for MLN8237 (Fig 4D) and VX680 (Fig S3C) were significantly reduced in sub-lines in which *HMMR*/RHAMM was silenced. Moreover, the levels of AURKA and p-AURKA immunofluorescence intensity were both augmented at spindle poles in mitotic 2884 cells with stable shRNA-mediated silencing of RHAMM (Fig. 4E). These findings suggest that silencing of *HMMR*/RHAMM augments AURKA activity and sensitizes MPNST cells to AKI.

**Figure 5:**
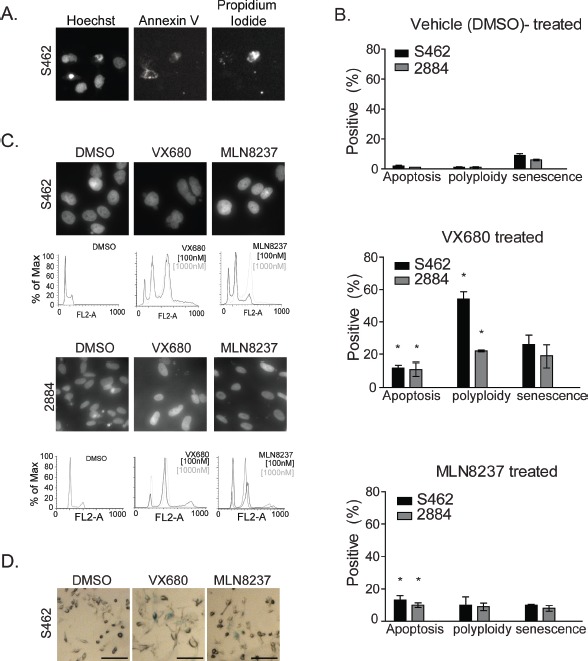
AKI treatment results in growth arrest, apoptosis, polyploidy and cellular senescence in MPNST cell lines *A*. Treatment of S462 and 2884 cells with AKI for 48 hours resulted in low levels of apoptosis as detected by Annexin V staining by high content cell screening. Representative images are shown with non-viable, membrane permeable cells counter stained with propidium iodide (PI). *B.* Quantitation of cellular responses to AKI or vehicle treatments, *p value <0.05, error bars = SD, n=3 for apoptosis measurements, n=3 for polyploidy and n=4 for senescence quantitation. *C*. S462 and 2884 cells were exposed to AKIs, or vehicle control, and changes in the nuclear content of treated cells was detected by PI staining and FACS analysis. S462 cells exposed to AKIs display larger G2/M and 8N fractions, indicative of an endoreduplication phenotype. Representative images of cells stained with DAPI are shown above the FACS profiles to illustrate the multi-nucleate phenotypes. *D*. Treatment of S462 cells with AKIs at IC-50 concentrations for 72 hours induces senescence activated β-gal expression in comparison to DMSO treated cells. Representative images were taken on an Axiovert 40 microscope (Zeiss).

We sought to identify and modulate the responsible pathways behind the cytostatic effects of MLN8237 on MPNST cell-lines and focused our initial investigations on pathways that are frequently altered in cells treated with AKI, such as apoptotic cell death, arrest at the G2-M transition, endoreduplication, and replicative senescence [8]. Through high-content analysis of nuclei that stained positive for annexin V (marker for early apoptosis) but not for propidium iodide (PI, marker for necrotic death), we found that after 48 or 72 hours (not shown) of MLN8237 treatment approximately 10% of cells were apoptotic regardless of the MPNST cell-line examined (Fig. 5A and 5B). Similar levels of apoptosis were also seen with exposure to VX680, which suggested that this cytostatic pathway may not account for the differential susceptibility of 2884 and S462 cells to these AKIs. Next, we examined the levels of polyploidy, as detected by immunofluorescence (DAPI counterstain) or fluorescent cytometry (PI) (Fig. 5C), and senescence, as measured through the activity of senescence activated beta-galactosidase [21] (Fig. 5D). After 72 hours exposure to VX680, but not to MLN8237, we found dramatic changes in gross nuclear morphology and the induction of senescence in both cell lines (Fig. 5B). Polyploidy was significantly more pronounced in S462 cells treated with VX680. However, it was only when the dose of MLN8237 was increased to 1μM that the levels of polyploidy observed by cytometry were significantly greater than vehicle-treated populations (Fig. 5C).

### MLN8237 induces the differentiation of MPNST cancer stem-like cells

**Figure 6:**
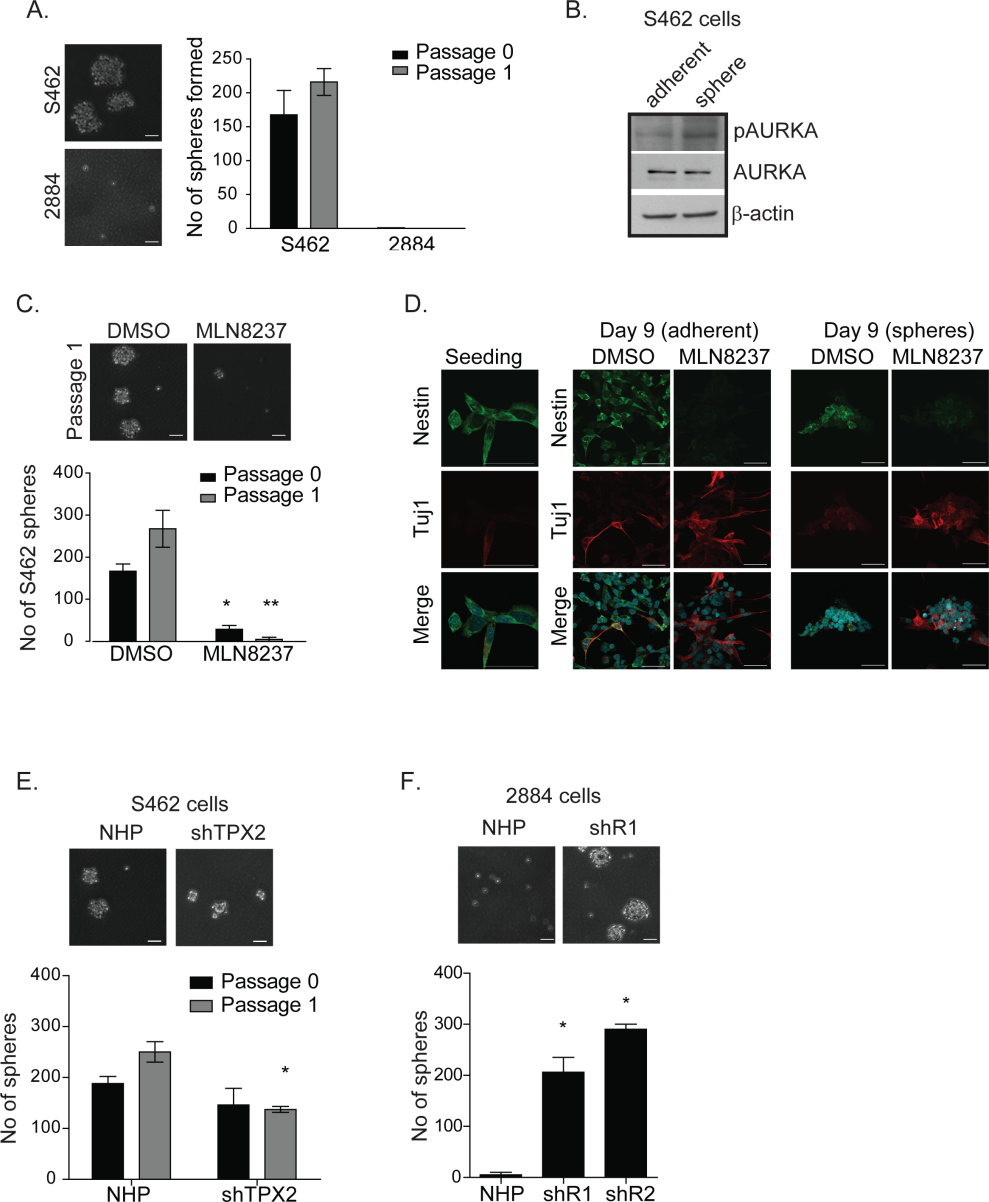
Inhibition of AURKA by MLN8237 limits self-renewal and induces neuronal differentiation of MPNST tumour initiating cells in vitro A. S462 and 2884 cells were cultured in anchorage independent conditions in neurocult media and sphere formation was quantitated after 6 days (passage 0). Spheres were dissociated and cultured for a further 6 days and sphere formation was measured again (passage 1). S462 cells form spheres whereas, 2884 cells do not. Representative images on the left, scale bars = 50μm, quantitation on the right, error bars = SD, n=3. *B*. In comparison to adherent S462 cell lysates, sphere-enriched S462 lysates (sphere) contain higher levels of auto-activated pAURKA (Thr288) indicating increased AURKA activity in sphere versus adherent S462 cells. β-actin serves as a loading control. *C.* In comparison to DMSO (vehicle) treated controls, prolonged treatment of S462 with 100 nM MLN8237 inhibits the propagation (passage 0) and self-renewal (passage 1) of S462 cells cultured as spheres. Quantitation is shown below representative images. Scale bars equal 50 μm. Error bars = SD, n=3, *p<0.05, **p<0.001. *D.* Dissociated sphere-enriched cells at passage 4 were fixed and stained for DAPI, nestin, and Tuj1 at day 0 and following 9 days of treatment with MLN8237 (50nM), or DMSO. Cells grew in both adherence and sphere phenotypes. While untreated cells maintain expression of nestin through the nine days of culture, MLN8237 treated spheres lost nestin expression and are positive for the neuronal marker, Tuj1 instead. Scale bars equal 50μm. *E.* shTPX2 and NHP S462 cells were grown in neurocult media. While there were no significant differences in sphere formation in passage 0, TPX2 knockdown is effecting the self-renewal of these spheres in passage 1, scale bars = 50μm, *p-value< 0.05, error bars = SD, n=3. *F*. shR1 and shR2 cells form significantly more spheres in neurocult media than NHP 2884 cells, indicating a role for RHAMM in sphere formation by modulating AURKA activity. Scale bars = 50μm, *p-value < 0.05, error bars = SD, n=3.

The levels of polyploidy, apoptosis or senescence did not explain the elevated sensitivity of S462 cells to MLN8237, so we investigated an alternative cytostatic pathway. As the S462 cell-line contains a subset of cancer stem-like cells [22] and small-molecule AKIs modify the cellular fates of mESCs and induced pluripotent stem cells [18, 19], we asked whether treatment with MLN8237 may promote the differentiation of these MPNST cancer stem-like cells. As a first step, we investigated the abilities of S462 and 2884 cell-lines to form spheroids when cultured under stem cell conditions. While no spheroids were identified in 2884 cultures, 5.6 ± 1.2% of S462 cells (168 ±25 of 3,000 seeded) are capable of forming spheroids (Fig. 6A). Secondary passage formed significantly more spheres (Fig. 6A, labeled passage 1), which is consistent with a published report describing self-renewal of sphere-initiating cells in these cultures [22]. Next, we measured AURKA activity and found that p-AURKA, but not total AURKA, was elevated in dissociated sphere cells relative to parental adherent cells (Fig. 6B). To determine whether AURKA activity was necessary for the growth or self-renewal of MPNST spheres, S462 cells were seeded at low densities in the presence of either DMSO or IC-50 concentrations of MLN8237. By Day 6 (passage 0), sphere formation was significantly inhibited in cells treated with MLN8237 in comparison to the vehicle alone treated cells (166 to 29 spheres in the DMSO and MLN8237 treated cells, respectively. p<0.05) (Fig 6C). When we dissociated spheres and re-seeded for a second passage, we found that the number of spheres formed was significantly reduced in the presence of MLN8237, which suggests that AURKA activity is necessary for the propagation and self-renewal of sphere-enriched cancer stem-like cells.

Compared to vehicle treated cultures, spheres that formed in the presence of AKI were significantly smaller in size and less uniform in shape (Fig. 6C), which suggested to us the potential engagement of a differentiation pathway. To determine whether treatment with MLN8237 can influence the differentiation capacity of MPNST cells, we dissociated sphere-enriched cells and cultured them on matrigel for nine days in the presence of MLN8237 or vehicle control. Dissociated sphere cells then grew either as adherent colonies or as attached spheres on the matrigel-coated coverslips (Fig. 6D). At seeding and after nine days of culture, sphere cells were stained for nestin, a type IV intermediate filament protein and marker for neuroprogenitor cells, and Tuj1, a neuron-specific class III β-tubulin and neural differentiation marker [23]. We found that MLN8237 treatment, in relation to vehicle alone, modified the cellular fate of sphere cells and resulted in the loss of nestin staining, strong expression of the neuronal marker Tuj1, and morphological changes consistent with neurons, such as neurite-like extensions (Fig. 6D). In contrast, DMSO vehicle treated control cells maintained a round morphology and nestin expression, suggesting that adherent culture alone was not inducing their differentiation.

To investigate whether silencing of *TPX2* or *HMMR*/RHAMM expression, respectively, is sufficient to prevent or endow sphere-forming capacity on MPNST cell-lines, we cultured the S462 and 2884 sub-lines under stem cell conditions. Stable silencing of *TPX2* did not significantly alter the propagation of sphere-forming S462 cells in the first passage, but may have impacted their self-renewal capacity, as indicated by a significant reduction in the number of spheres formed during the second passage (Fig. 6E). However, the stable silencing of *HMMR*/ RHAMM endowed 2884 cells with the capacity to both propagate and self-renew as sphere-forming cells (Fig 6F).

## DISCUSSION

A recent study identified AKI as a viable treatment platform for MPNST by first modeling the disease in an animal model and then identifying differentially expressed genes, including *AURKA* [5]. In common with their report, we identified genomic amplification of *AURKA* in MPNST cell-lines (Fig S2B) and found that robust inhibition of growth *in vitro* followed the silencing of *AURKA* or the treatment of cells with AKI, which also stabilized the growth of two primary, human MPNST when grown as xenotransplants in animal models. By studying the responses of primary human MPNSTs grown *in vivo*, our animal models also contain non-neoplastic cells from the human tumour environment, which are critical for tumourigenicity [24]. However, we did not identify a significant level of endoreduplication downstream of AKI except at relatively high doses *in vitro* and within our treated tumours *in vivo*, which may reflect concomitant inhibition of AURKA and AURKB at these dosages. Mitotic failure and polyploidy are common cellular response to inhibition of AURKB [8] and both MLN8237 *in vivo* treatment protocols (20 mg/kg/dose, twice daily or 30 mg/kg/dose, one daily) result in blood plasma levels in excess of 2.5 μM [5, 7], which exceeds the described IC-50 against AURKB in cell-based assays [7]. We found, however, that a subset of sphere-enriched MPNST cells may engage a differentiation program in response to AKI.

To our knowledge, this is the first reported use of AKI to drive the differentiation of sphere-enriched cancer stem-like cells. We found that AURKA activity is critical to the propagation and self-renewal of MPNST sphere cells and that these phenotypes were responsive to the silencing of regulators for the kinase, such as *TPX2* and *HMMR*/RHAMM. A strong correlation has been drawn between AURKA expression and the maintenance of pluripotency in murine embryonic stem cells (mESCs) and, similar to our findings, silencing *AURKA* and treatment with AKI was sufficient to drive their differentiation [18]. In mESCs, this action of AURKA relies upon the phosphorylation and suppression of p53 activity, which may be a TPX2-independent action as silencing *TPX2* did not cause significant defects in self-renewal [18]. In our studies, however, we find that altering the abundance of either TPX2 or *HMMR*/RHAMM was sufficient to modify the growth of MPNST cells as spheres in stem cell cultures. Thus, mitotic and non-mitotic roles for AURKA are likely to regulate the self-renewal and differentiation of sphere-enriched cancer stem-like cells, mESC self-renewal [18], and reprogramming of somatic cells to produce human induced pluripotent stem cells [19].

MPNSTs often resist chemotherapies and are prone to high rates of disease relapse [2-4]. Here, we find that AKI may potentiate the growth and self-renewal of MPNST spheroid forming cells, which suggests that these drugs may be effective against recurrent disease arising from a reservoir of cancer-stem like cells. Indeed, inhibitors for aurora kinases are effective against tumour cell lines that are refractory to EGFR inhibition [25, 26]. These cellular responses to AKI likely rely upon the activity of p53, which is often lost in MPNST [27] and is critical to the supervision of mitotic failure and tetraploidy [28, 29]. AKIs are sufficient to delay G2/M progression and the resolution of this delay, be it apoptosis or mitotic slippage followed by endoreduplication, relies upon the action of p53 [30]. Therefore, concurrent activation of the p53 pathway may be an important consideration when designing combination therapies for AKIs against MPNST. Such an approach has been shown to augment the pro-apoptotic actions of AKIs in cell-line models for carcinomas [31, 32].

Molecular-targeted single agent therapies often fail to demonstrate a significant survival benefit in patient populations in part due to our inability to predict tumour responses. So, we aimed to understand and modulate the response of MPNST to AKI. Our study shows that the responses of MPNST cells *in vitro* to AKI treatment can be significantly altered by the silencing of key regulatory molecules. For example, silencing of *HMMR/*RHAMM in 2884 cells, which contain genomic amplification of 5q32-qter, significantly impacts the sensitivity to AKI and endows these cells with the ability to grow in stem cell culture, presumably through the release of TPX2 and activation of AURKA. Consistently, silencing of *HMMR*/RHAMM in breast epithelia cells modulates TPX2 location and augments kinase activity [11] while attenuation of *Xenopus* RHAMM (XRHAMM) may activate AURKA during anastral spindle assembly [33]. Genomic changes that accompany the transition of benign neurofibromas to MPNSTs occur at the *HMMR*/RHAMM locus [9], the *AURKA* locus [5](Fig. S2B) and chromosome 20q (containing *AURKA* and *TPX2*) [9, 15, 16]. Therefore, these genomic imbalances may endow a growth advantage to a subset of human primary MPNSTs but also make these tumours exquisitely sensitive to AKI.

Silencing of RHAMM may affect AURKA activity in a context or cell-type dependent manner. In multiple myeloma cells, RHAMM depletion protects against AKI treatment [34]. In Xenopus, XRHAMM depletion impaired TPX2 location and anastral assembly [13], which should reduce AURKA activity at sites of assembly. We believe these apparent contradictory findings are resolved by a bimodal model for RHAMM function. RHAMM is critical to the correct mitotic location of TPX2 [13, 14] and, therefore, its complete loss impairs TPX2-AURKA complex formation and attenuates kinase activity. However, RHAMM is also a substrate for the active kinase that negatively regulates AURKA activation through the sequestration of TPX2 [11]. Thus, the hemizygous loss of *HMMR*/RHAMM, or silencing its expression in 2884 cells with genomic amplification of 5q, may relax this negative feedback mechanism, augment kinase activity, and sensitize tumour cells to AKI.

Our studies indicate that hemizygous loss of *HMMR*/RHAMM, which occurs in approximately 50% of aggressive MPNST [9], may oncogene-addict tumours to AURKA activity and sensitize these cancers to AKI. This insight may be applied to other tumour subtypes. For example, variation in the genomic region surrounding *HMMR/*RHAMM, and the *HMMR*/RHAMM gene [11], modifies the risk to develop breast cancer in carriers of germline *BRCA1* mutations [35]. These carriers often develop breast tumours that do not express estrogen receptor, progesterone receptor or human epidermal growth factor receptor (HER-2), resemble basal epithelia by gene expression profiles (i.e. basal subtype), and are refractory to anti-estrogen therapy, hormone therapy, and targeted therapies directed against HER-2 [36]. The loss of 5qter, a genomic region surrounding *HMMR*/RHAMM, is a frequent event in basal subtype breast cancer [37], and this genomic loss associates with an increase in the expression of AURKA in trans [38]. Thus, genomic loss of *HMMR*/RHAMM may indicate a subset of tumours that are reliant upon AURKA and highly susceptible to AKI.

In summary, our findings and those of Patel and colleagues [5] highlight AURKA as a new therapeutic target for MPNST. For the first time, we describe a pro-differentiation effect for AKI on a subset of sphere-enriched cancer stem-like cells and demonstrate that copy number alteration of regulatory gene-products, like *HMMR*/RHAMM and TPX2, can significantly impact the tumour cellular responses to these emerging therapies.

## MATERIALS AND METHODS

### *In vivo* animal models

Four-month-old male *NOD/SCID* mice were anesthetized with isofluorane and orthotopically implanted with 1 mm^3^ piece of explant tumour in both legs, near the sciatic nerve (Castellsagué et al. manuscript under preparation). Tumour volume was calculated at V= (W^2^*L (π/6)), where L is the longest diameter and W is the width. Mice were randomly assigned to treatment or control groups. Dosage of MLN8237 in mice was previously determined (31). Mice were treated by gavage once daily with 30mg/kg MLN8237 or vehicle (10% 2-hydroxypropyl-β-cyclodextrin and 1% sodium bicarbonate) for 28 days. Tumours were measured every 3 days. At the end of the treatment period, mice were euthanized and harvested tumours were fixed in formalin.

### Adherent culture and culture as spheres

Cell-lines were obtained from Drs. VF Mautner and L Kluwe (University Hospital Eppendorf, Germany) or the ATCC and were cultured as recommended. Unless noted otherwise, cells were seeded at 1.0 × 10^4^ (S462 cell-line) or 3.0 × 10^4^ (2884 and 2885 cell-lines) cells/well in 24-well culture plates and allowed to adhere for 24 hours prior to the denoted treatment. These seeding densities resulted in equivalent growth kinetics for S462 and 2884 cells. The culture of S462 spheres followed the protocol in [22].

### siRNA and small molecule reagents

siRNA (Qiagen, Table S1) were transfected at 10 pmol/well using Lipofectamine2000™ according to manufacturer's protocols (Invitrogen). AURKA knockdown was confirmed at 48 hours post transfection. Small-molecule AKIs were dissolved and diluted in DMSO/DMEM and included the pan-aurora inhibitors C1368 (Sigma) and VX680 (Selleck Chemicals), and the AURKA specific inhibitor MLN8237 (Selleck Chemicals).

### Lentivirus mediated shRNA knockdown and generation of sub-lines

Lentivirus packaging, envelope and control non-hairpin (NHP) pLKO.1 plasmids (Addgene) were used with shRNA against RHAMM and TPX2 (Sigma) as described in [11] (Table S1). Transfected cells were selected with 0.5μg/ml puromycin (GIBCO) and maintained with 0.3μg/ml puromycin.

### Immunofluorescence, immunohistochemistry, and immunoblot analyses

Antibodies were sourced as follows: RHAMM (Epitomics), TPX2 (Novus), nestin (Covance), Tuj1 (Covance), β-actin (Sigma), AURKA, phospho (p)-AURKA (Thr288), p-histone H3 (Ser10), and caspase 9 (Cell Signalling). The p-RHAMM (Thr703) polyclonal antibody is characterized in [11].

Cell-lines were fixed and permeabilized in methanol. Antibodies were diluted in PBS-0.1% Tween and 3% BSA (Sigma). Coverslips were mounted in 90% glycerol/PBS with DAPI (Invitrogen) and images were acquired and analyzed using an Olympus FV10i confocal microscope.

For immunofluorescence of spheres, coverslips were coated with 2% geltrex (Invitrogen) in Neurocult media (StemCell Technologies) before cells were plated. Cells were fixed with 4% PFA and then stained.

Immunohistochemistry on paraffin sections was done as in [11]. Secondary antibody staining was done with the relevant antibodies for 30 minutes at room temperature. Staining was visualized by 3,3-diaminobenzidine, with Hematoxylin as a counter-stain.

Western blot analyses were performed on lysates collected from sub-confluent MPNST cells lysed in modified RIPA buffer, as described [39]. Levels of protein were detected and quantified with the Odyssey infra-red imaging system (LI-COR) using IRDye 800- or IRDye 680-conjugated secondary antibodies (Rockland) or through chemiluminescence detection of HRP-conjugated antibodies (Sigma).

### Genomic and reverse-transcriptase PCR and real-time PCR

Genomic DNA was extracted with the DNeasy extraction kit (Qiagen) and preparations were measured with a NanoDrop (Thermo-Fisher). For real-time, genomic PCR, reactions were run in triplicate with an Applied Biosystems 7000 series machine (Invitrogen). RNA was extracted using the RNeasy kit (Qiagen), quantified with NanoDrop and converted to cDNA using AccessQuick (VWR) as per manufacturer's protocols. For primers and PCR conditions see Table S1. Results were analyzed using the ΔΔCt method. Expression of transcript/gene was normalized to TATA box binding protein levels, which was then normalized to levels of transcript/gene in 2885 cells.

### Array comparative genomic hybridization (a-CGH)

Genomic DNA was isolated using the Gentra Puregene Cell Kit (Qiagen). Purified DNA was digested with Bovine DNase I (Ambion). Test and control samples were labelled with Alexa5 and Alexa3 dyes respectively using the BioPrime Total Genomic Labeling Kit (Invitrogen). Labeled samples were competitively hybridized to 2 × 400k Human CGH Arrays (Agilent Technologies) as recommended by the manufacturer. Copy number estimates were extracted from the microarray image files using Feature Extraction 10.5 (Agilent) and the data were analyzed in Agilent Genomic Workbench 6.5 (Agilent) after centralization and fuzzy zero normalization matrixes were applied. Copy number abnormalities were identified using the ADM-2 algorithm with a threshold setting of 5.5 and regions were only considered significant if they were defined by a minimum of 3 probes and the average log_2_ value exceeded a threshold of 0.2 (grey shaded regions).

### Cell based assays

For cell viability assays, MPNST cell-lines were plated and after 24 hours were treated with carrier alone (0.1% DMSO) or AKIs at indicated concentrations. Viability was quantified after 72 hours by addition of 5 mg/ml 3-(4,5-dimethylthiazol-2-yl)-5-(3-carboxymethoxyphenyl)-2-(4-sulfophenyl)-2*H*-tetrazolium (MTT, Invitrogen) as recommended by the manufacturer. Each condition was done in triplicate and repeated three times. For growth curves, MTT assays were conducted at 24, 48, 72 and 96 hours after plating.

For nuclear content analysis by FACS, cells were harvested, fixed and stained as in [40] and analyzed with FACS Calibur and CellQuestPro software (BD Biosciences), respectively. For polyploidy analysis, AKI treated cells were stained with DAPI. Nuclei area was measured using Image J software. Annexin V (BD Biosciences) staining followed manufacturer's protocol. Images were collected with a High Content Analyzer (ArrayScan VTI, Cellomics).

Cellular senescence was measured by detection of senescence-activated β-Galactosidase activity. Cells were plated, treated with AKIs for 72 hours, fixed and stained as described in [21].

### Statistics

Statistical significance was evaluated by unpaired two-tailed Student's t-tests with p>0.05. Two way ANOVA was used to determine significance between treatments for *in vivo* tumour volumes using GraphPad Prism.

## Supplementary Figures and Tables


