# Supplementary material for: Genomic imbalance of HMMR/RHAMM regulates the sensitivity and response of malignant peripheral nerve sheath tumour cells to aurora kinase inhibition
Source: Oncotarget. 2013 Jan 9;4(1):80–93. doi: 10.18632/oncotarget.793 (PMC3702209; doi:10.18632/oncotarget.793)
Supplement: Supplementary file 1 [file oncotarget-04-080-s001.pdf]

Genomic imbalance of HMMR/RHAMM regulates the sensitivity and response of malignant peripheral nerve sheath tumour cells to aurora kinase inhibition.

Supplemental Material

Table S1: PCR conditions, primers, shRNA and siRNA in MPNST cell lines.

Genomic DNA targeted primers span intro-exon boundaries.

**Gene**	**Target**	**Region**	**Primer pair**
HMMR	Genomic	Intron 9-Exon 10	5’ GCTGAAAGGCTGGTCAAGC 3’<br>5’CCAACCTAACACGCTCACAT 3’<br><br>5 cycles at 95°C for 5 minutes, 60°C for 60s and 72°C for 60s, and 35 cycles at 95°C for 60s, 60°C for 30s and 72°C for 30s (for all qPCRs)
	Message	Exon 9-Exon 10	5’ TGTGCTTCAGATCAAGTGG 3’<br>5’ CGTTGTGTTCTCTATTCCTG 3’<br><br>95°C for 60s, 60°C for 30s and 72°C for 30s for 35 cycles (for all qRT-PCRs)
TPX2	Genomic	Exon 1-Intron 2	5’ AAACCACAGGTAAGGCAGTGAC 3’<br>5’ TCACCCACTATCCCACCTCT 3’
	Message	Exon 5	5’ AGCCTTTCAACCTGTCCCAAGGA 3’<br>5’ AGACAGGGTCTTGCTCCGTCA 3’
RHAMM	shRNA		5’ CGTCTCCTCTATGAAGAACTA 3’<br>5’ GCCAACTCAAATCGGAAGTAT 3’
TPX2	shRNA		5’ CCGAGCCTATTGGCTTTGATT 3’
AURKA	siRNA		5’TCCCAGCGCATTCCTTTGCAA 3’<br>5’ CAGGGCTGCCATATAACCTGA 3’<br>5’ CACGTGCTCTACCTCCATTTA 3’<br>5’ CACCTTCGGCATCCTAATATT 3’

Supplementary Figure Legends

Supplementary Figure 1: Aurora kinase inhibitors are effective in decreasing kinase activity. *A.* S462 cells were treated with 1x (100 nM) and 10x IC-50 (1000nM) doses of MLN8237 and VX680 inhibitors. Immunoblot analysis shows that inhibition of the kinase reduced the levels of p-RHAMM in a dose-dependent manner after 3 hours, while overall levels of Aurora A and RHAMM remain stable with treatment. β-actin serves as a loading control. *B.* VX680- treated HeLa cells also have reduced levels of the active kinase (p-Aurora (Thr288)) and two substrates (p-RHAMM (Thr703) and p-Histone H3(Ser10)) as detected by immunofluorescence. Scale bars represent 5 μm. *C.* MTT assays indicate different IC-50s for the three different inhibitors, with MLN8237and VX680 being the most potent while a commercially available inhibitor, termed C1368, is less potent. MPNST cells were treated with increasing doses of AKIs and cell viability was measured after 72 hours. Error bars = SEM, n=3.

Supplementary Figure 2: Comparative genomic hybridization (CGH) in MPNST cell lines show amplifications in *AURKA*. *A.* CGH of the entire genome of the MPNST cell lines 2885, 2884 and S462. *B*. CGH of the *AURKA* locus highlighting the amplification of this region in both the S462 and 2884 cell lines but not the 2885 line.

Supplementary Figure 3: Growth kinetics and the effect of VX680 on shTPX2, shR1 and shR2 lines. *A.* S462 cells with shRNA mediated silencing of TPX2 have similar proliferation rates to their non-hairpin (NHP) controls. Cell viability was measured by MTT assays at 24, 48, 72 and 96 hours and normalized to Day 1. Error bars = SD. *B*. IC-50s of VX680 treatment on

shTPX2 and NHP S462 cells. Cell viability was measured after 72 hours of VX680 treatment at various concentrations by MTT assays. Error bars = SD, n=3. *C.* Growth kinetics of shR1, shR2 and NHP transfected 2884 cells were not significantly different. Cell viability was measured by MTT assays at 24, 48, 72 and 96 hours and normalized to Day 1. Error bars = SD. *D.* IC-50s of VX680 treatment on shR1, shR2 and NHP 2884 cells. Cell viability was measured after 72 hours of VX680 treatment at various concentrations by MTT assays, p value <0.05, error bars = SD, n=3.

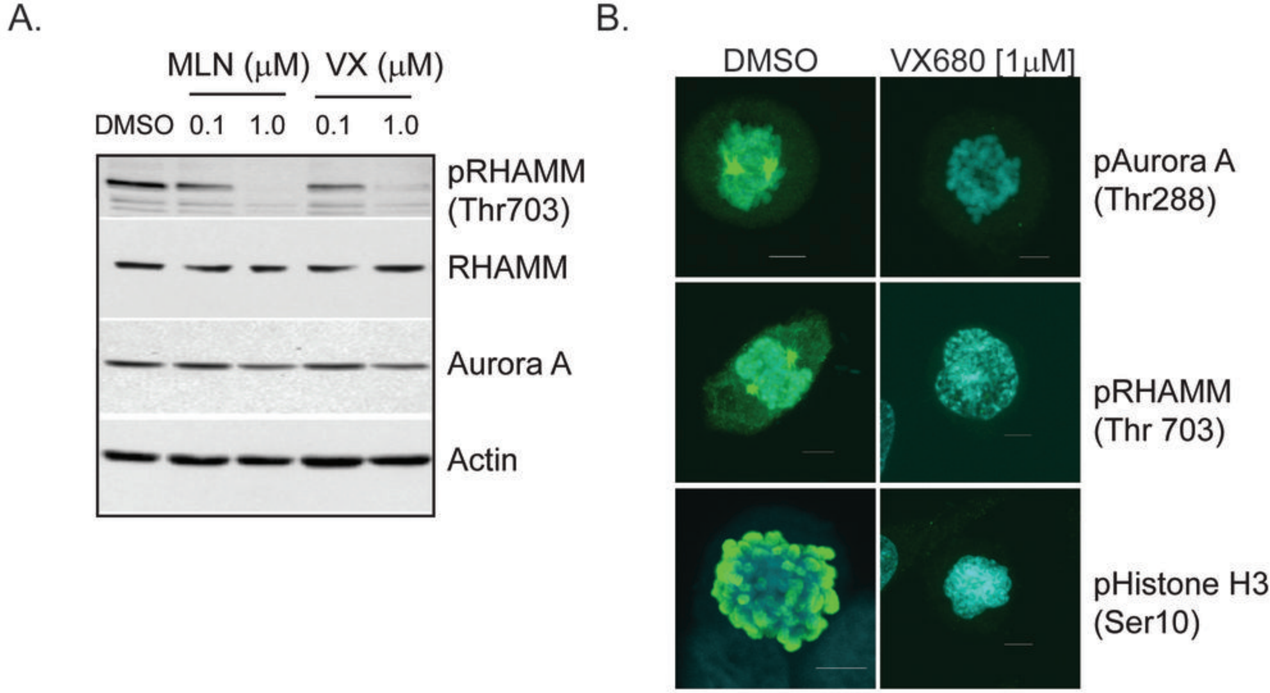
A.
MLN (μM)
VX (μM)
DMSO
0.1
1.0
0.1
1.0
pRHAMM (Thr703)
RHAMM
Aurora A
Actin
B.
DMSO
VX680 [1μM]
pAurora A (Thr288)
pRHAMM (Thr 703)
pHistone H3 (Ser10)

Figure S1

A.

B.

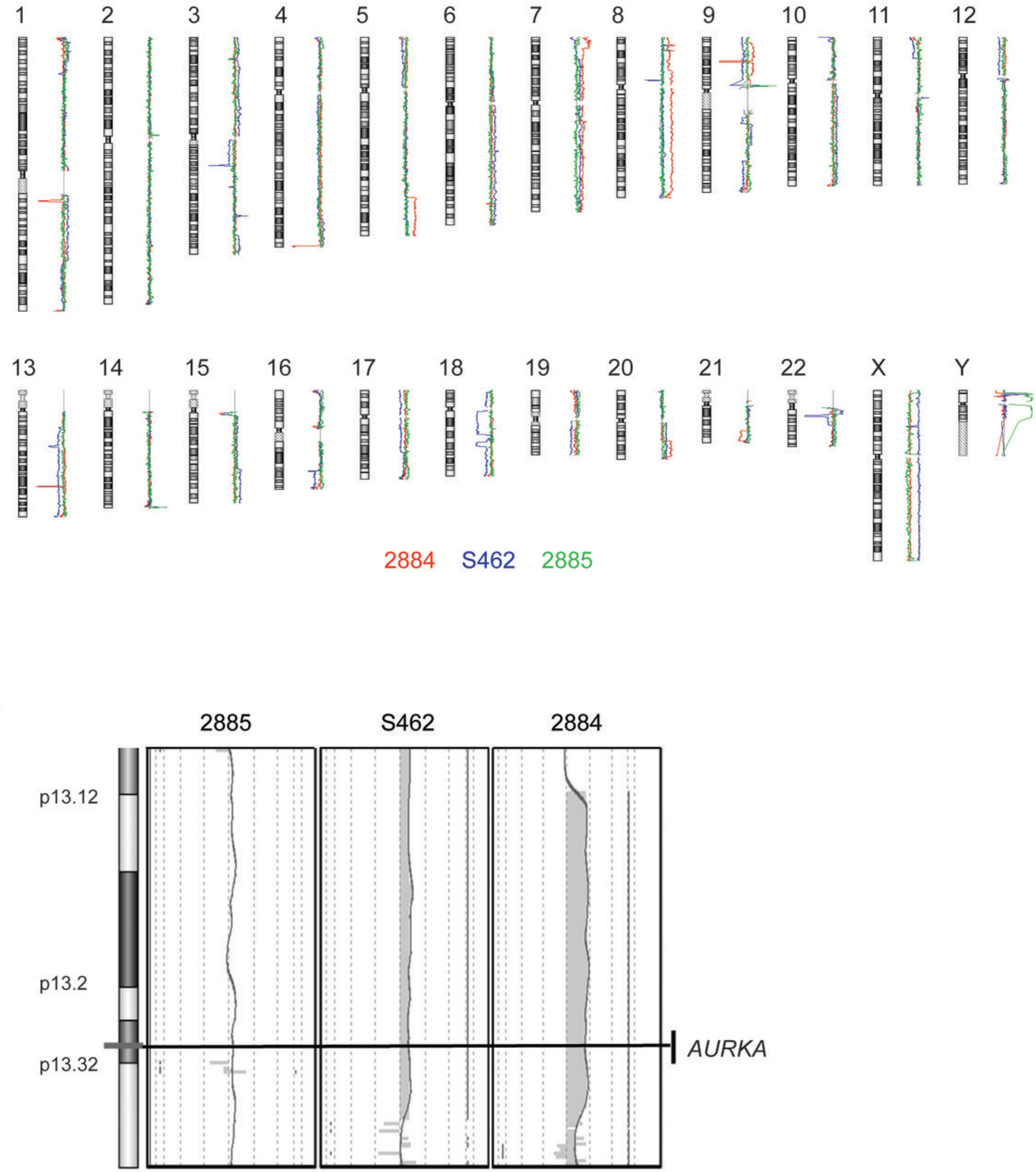

1
2
3
4
5
6
7
8
9
10
11
12
13
14
15
16
17
18
19
20
21
22
X
Y
2884
S462
2885
2885
S462
2884
p13.12
p13.2
p13.32
AURKA

Figure S2

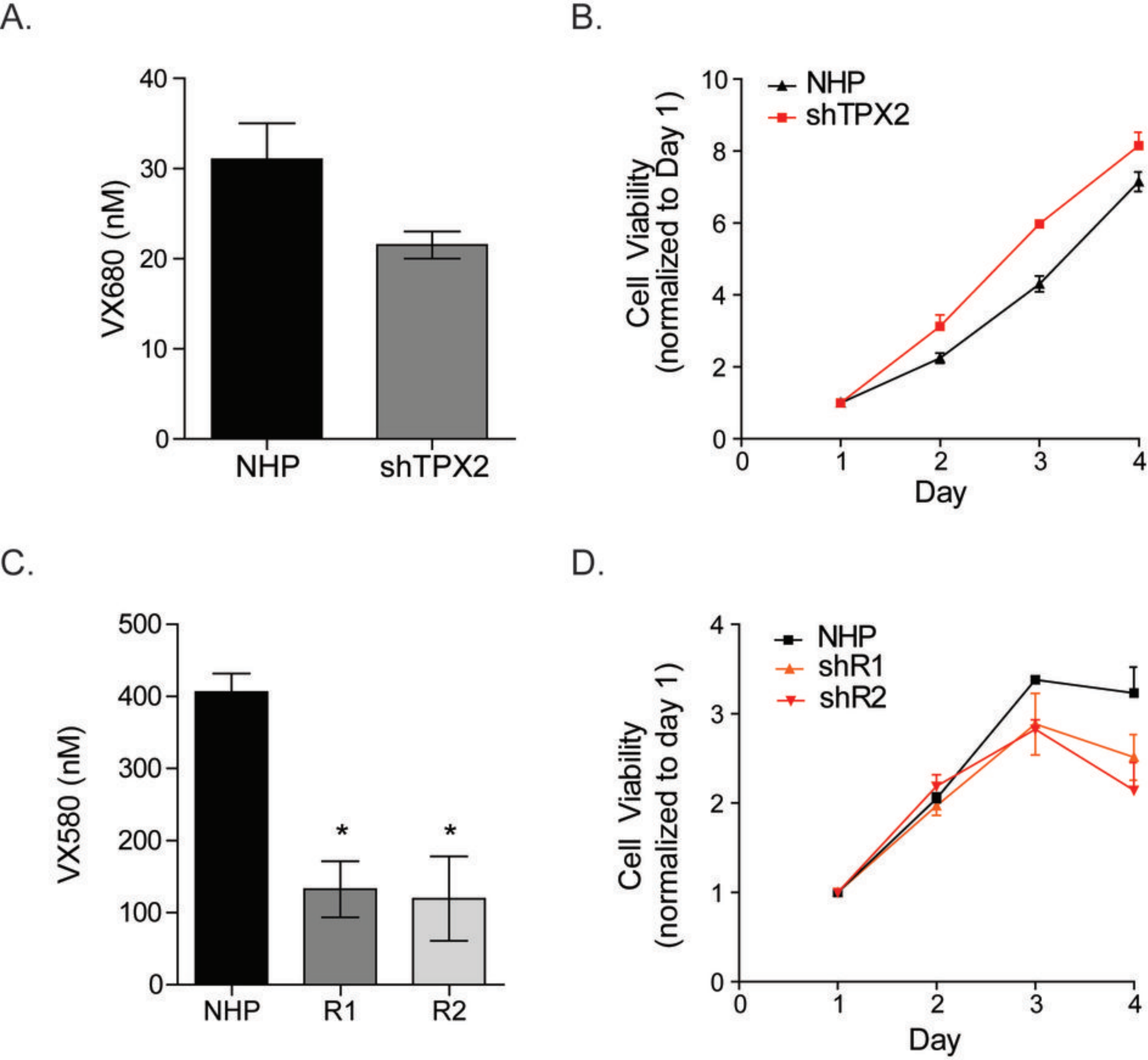
A.
VX680 (nM)
NHP
shTPX2
B.
Cell Viability (normalized to Day 1)
NHP
shTPX2
Day
C.
VX580 (nM)
NHP
R1
R2
D.
Cell Viability (normalized to day 1)
NHP
shR1
shR2
Day

Figure S3
